# Selective sweep on human amylase genes postdates the split with Neanderthals

**DOI:** 10.1038/srep37198

**Published:** 2016-11-17

**Authors:** Charlotte E. Inchley, Cynthia D. A. Larbey, Nzar A. A. Shwan, Luca Pagani, Lauri Saag, Tiago Antão, Guy Jacobs, Georgi Hudjashov, Ene Metspalu, Mario Mitt, Christina A. Eichstaedt, Boris Malyarchuk, Miroslava Derenko, Joseph Wee, Syafiq Abdullah, François-Xavier Ricaut, Maru Mormina, Reedik Mägi, Richard Villems, Mait Metspalu, Martin K. Jones, John A. L. Armour, Toomas Kivisild

**Affiliations:** 1 Department of Archaeology and Anthropology, University of Cambridge, Cambridge, CB2 3QG, UK; 2 School of Life Sciences, Queen’s Medical Centre, University of Nottingham, Nottingham NG7 2UH, UK; 3 Scientific Research Centre, University of Salahaddin, Erbil, Kurdistan, Iraq; 4 Estonian Biocentre, Tartu, 51010, Estonia; 5 Division of Biological Sciences, University of Montana, Missoula, MT, USA; 6 Complexity Institute, Nanyang Technological University, Singapore; 7 Statistics and Bioinformatics Group, Institute of Fundamental Sciences, Massey University, Palmerston North, New Zealand; 8 Estonian Genome Center, University of Tartu, Tartu, Estonia; 9 Department of Biotechnology, Institute of Molecular and Cell Biology, University of Tartu, Tartu, Estonia; 10 Thoraxclinic at the University Hospital Heidelberg, 69126 Heidelberg, Germany; 11 Genetics Laboratory, Institute of Biological Problems of the North, Russian Academy of Sciences, Magadan, Russia; 12 Division of Radiation Oncology, National Cancer Centre, Singapore; 13 RIPAS Hospital, Bandar Seri Begawan, Brunei Darussalam; 14 Evolutionary Medicine group, Laboratoire d’Anthropologie Moléculaire et Imagerie de Synthèse, UMR 5288, Centre National de la Recherche Scientifique, Université de Toulouse 3, Toulouse, France; 15 Department of Applied Social Sciences, University of Winchester, Sparkford Road, Winchester SO22 4NR, UK; 16 Department of Evolutionary Biology, Institute of Molecular and Cell Biology, University of Tartu, 51010, Estonia; 17 Estonian Academy of Sciences, 10130 Tallinn, Estonia

## Abstract

Humans have more copies of amylase genes than other primates. It is still poorly understood, however, when the copy number expansion occurred and whether its spread was enhanced by selection. Here we assess amylase copy numbers in a global sample of 480 high coverage genomes and find that regions flanking the amylase locus show notable depression of genetic diversity both in African and non-African populations. Analysis of genetic variation in these regions supports the model of an early selective sweep in the human lineage after the split of humans from Neanderthals which led to the fixation of multiple copies of *AMY1* in place of a single copy. We find evidence of multiple secondary losses of copy number with the highest frequency (52%) of a deletion of *AMY2A* and associated low copy number of *AMY1* in Northeast Siberian populations whose diet has been low in starch content.

Alpha-amylases, hereafter referred to as amylases, are expressed in the salivary glands and pancreas by genes of the AMY gene family which in humans has a variable number of gene copies that co-localize as a tight cluster in a ~200 kb region on the reference assembly on chromosome 1. Copies of the *AMY1* gene are expressed in the salivary glands and allow pre-ingestion breakdown of carbohydrates into simple sugars. *AMY2* copies are expressed in the pancreas and serve the same purpose in the duodenum. Compared to other great apes the AMY locus is significantly expanded in humans, with high levels of copy number variation (CNV) found within and among populations^1^,^2^,^3^,^4^,^5^,^6^. Higher copy number of the *AMY1* gene has been shown by multiple studies to cause increased expression of amylase in the saliva which is likely to enable more efficient digestion of starch-rich foods^1^,^7^. However, the broader phenotypic effect of amylase CNV is still poorly understood as the initial reports for *AMY1* copy number association with body mass index and obesity risk^7^,^8^ have failed replication^4^.

It has been proposed that the *AMY1* CNV expansion may be recent and associated with positive selection during the shifts from the low starch diet of hunter-gatherers to the starch-rich diets of Neolithic farmers^1^. The inference of positive selection was an observation of significantly higher differentiation of *AMY1* copy number between high-starch diet Japanese and low-starch diet Yakutians, as compared to the genome-wide range of variation in other copy number and microsatellite loci. However, the qPCR method used in this earlier work to ascertain *AMY1* copy numbers has been shown to lack sufficient accuracy^3^,^4^. Concerns also remained about the assignment of populations into groups of historically high versus low starch consumers^9^. Furthermore, ancient DNA evidence shows that an 8,000 year old Mesolithic hunter-gatherer from Loschbour, Luxembourg, already carried 13 copies of the *AMY1* gene, which is at the high end of the variation observed in present-day Europeans^2^. This suggests that selection operating since the agricultural transition cannot fully explain the high number of amylase copies in humans. Despite the evidence for high differentiation between Japanese and Yakutian *AMY1* copy numbers, scans of long-range haplotype homozygosity have failed so far to highlight the amylase locus as a significant target of recent positive selection in representative groups of human populations^10^,^11^. These varied lines of evidence suggest a complex evolutionary narrative of the *AMY* locus in humans that is not entirely captured by the model of recent selection during the Holocene period associated with agricultural subsistence.

Amylase copy number amplification may have started early in human evolutionary history, in the Pleistocene period. Because the effectiveness of salivary amylase is significantly enhanced when digesting cooked rather than raw starches^12^ it has been suggested that multiplication of amylase genes would have become selectively advantageous, as an energy source to sustain the growing brain size, only after cooking had become widespread. Even though the earliest use of fire by hominins has been predicted at 1.9 MYA^13^, the earliest evidence for consistent and repeated use of fire hearths is more recent and starts to emerge in Middle Pleistocene, only ca 300 KYA^14^. The relationship between cooked food and hominin brain size has been the focus of extensive anthropological research^13^,^15^,^16^,^17^. Considering archaeological, anthropological and genetic evidence available so far Hardy *et al.*^9^ have hypothesized that the spread of cooking of starch-rich tubers in combination with the gain of higher *AMY1* copy numbers by our ancestors may have facilitated the rapid growth of brain size in the early Middle Pleistocene ca 800 KYA, before the split of modern humans and Neanderthals.

As both humans and Neanderthals are characterised by increased brain volumes at the species level, and given the potential overlap between the time depth of cooking and the divergence of these species, ancient DNA of extinct hominins provides a vital additional window into *AMY* evolution. While most humans today carry more copies of salivary *AMY1* genes, Neanderthals, Denisovans, and a small number of present-day Europeans have been found to carry the ancestral number of two *AMY1* copies per diploid genome^18^. With respect to the critical, unanswered questions as to when, where, and why amylase gene numbers increased, two possible scenarios can explain this: a) incomplete lineage sorting at the AMY locus, to explain the co-existence of low and high copy number lineages in two closely related species, and, early Middle Pleistocene amplification of *AMY1* copy numbers, as proposed by Hardy *et al.*, in the species that was ancestral to humans and Neanderthals to sustain the energetic needs of the growing brain and, potentially, secondary reduction of *AMY1* copy numbers in Neanderthals enabled by their switch to high protein meat diet (Hardy *et al.*^9^); or b) late Middle Pleistocene selective sweep at the *AMY1* locus after the human-Neanderthal split in conjunction with a divergence in associated food technologies and followed by secondary losses of *AMY1* variation in some human populations. Neither of these scenarios precludes later selection associated with agricultural subsistence.

Patterns of genetic variation accumulated at the *AMY* locus and its flanks ascertained in a range of human populations can be informative for resolving these different possibilities. While previous studies^1^,^3^,^4^ have provided us with the first insights into the global distribution of CNV in the *AMY* locus, large regions such as Siberia, South East Asia and the Americas are still relatively poorly covered by data. In this study we use high coverage sequence data from 480 individuals sampled across the world to search for evidence of either recent or old signals of positive selection at the amylase locus. We confirm the robustness of estimating *AMY1*, *AMY2A* and *AMY2B* gene copy number from read depth data by genotyping a subset of 68 samples with paralogue ratio tests and microsatellite assay methods. By assessing the diversity, age and affinity to archaic humans of haplotypes associated with high and low *AMY1* copy numbers we test whether the haplotypes associated with ancestral copy number in presently living humans can be explained by the retention of ancestral haplotypes, introgression from archaic hominins or convergence due to secondary deletions in the locus.

## Results

**Figure 1:**
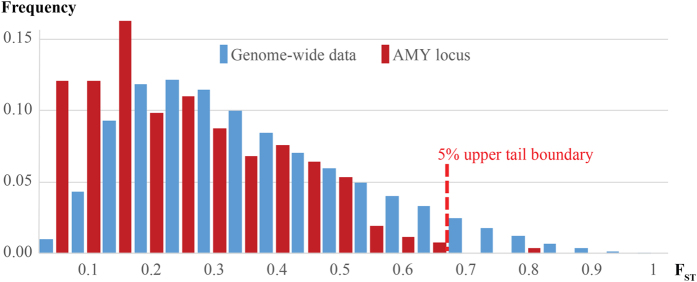
Distribution of estimates of maximum pairwise F_st_ per 200 kb non-overlapping segment among 12 human population groups. ‘AMY locus’ refers to data from three neighbouring 200 kb segments from the Chr1:103,800,000-104,400,000 region. The distribution of the maximum F_ST_ scores of the 200 kb regions is shown by bins of 0.05. The only 5% significant (red dotted line) F_ST_ estimate for the AMY locus comes from the African and Northeast Siberian comparison at the Chr1:104,000,000-104,200,000 segment.

To cast further light on the question of whether the *AMY* locus has been subject to recent positive selection in humans we first examined the extent of genetic differentiation of human populations at the *AMY* locus in the context of other genetic loci in a global data set of 480 high coverage genomes^19^ (Table S1). We compared the maximum *F*_*st*_ values of SNPs in 200 kb regions immediately surrounding the AMY locus against the distribution of similar values obtained from genome wide data and found that genomic regions flanking the amylase genes are characterized globally by a significant (χ^2^–test, p < 0.001) deficit rather than enrichment of high *F*_*st*_ values: only one pairwise comparison, involving Africans vs Northeast Siberians, yielded an *F*_*st*_ score within the top 5% genome-wide ranks (Fig. 1). Furthermore, the distribution of the maximum *F*_*st*_ values by 200 kb windows from *AMY* flanks showed a relatively higher (50%) proportion of estimates of low differentiation (*F*_*st*_ < 0.2) compared to the genome-wide average of 26%. These results are unexpected under the scenario of recent selective sweeps and, in particular, do not support the model of Perry *et al.*^1^ which would predict high *F*_*st*_ values between our Southeast Asian and Northeast Siberian groups. Furthermore, none of the populations we examined showed signal of recent positive selection at p < 0.01 by the two commonly used haplotype homozygosity tests nSL and iHS (Table 1).

**Figure 2:**
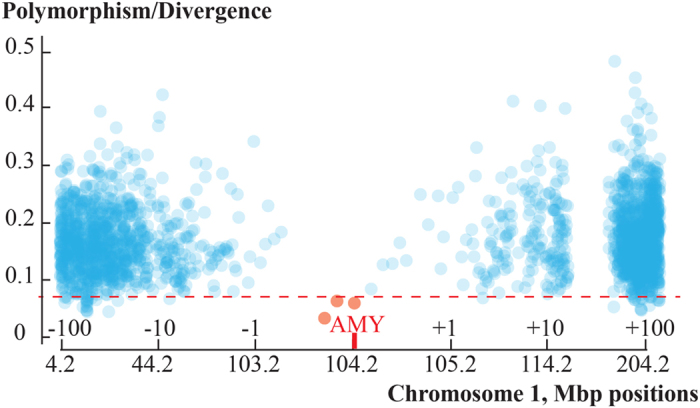
Distribution of genetic diversity on chromosome 1 in human populations. Presented on the y axis is the sum of derived allele frequency over all polymorphic loci in 1000 Genomes African data, estimated by 50,000 bp non-overlapping segments of chromosome 1, relative to the divergence of the human reference sequence from the ancestral sequence (determined by the 6 primate sequence consensus). Red dotted line indicates the 1% cut-off considering the empirical genome-wide distribution. Only those 50,000 bp segments which had >90% sites covered in human, Altai Neanderthal and Denisovan data were considered.

To explore the possibility of an older selective sweep we used Tajima’s D^20^ test which highlighted the *AMY* region in Africans as a significant candidate of positive selection (p = 0.0024; Table 1). To further assess the magnitude of the signal revealed by the Tajima’s D test we scanned the genomes of the global set of 480 individuals by 50 kb non-overlapping windows for cumulative frequency of derived alleles at sites polymorphic in humans. We found that the 50 kb regions both upstream and downstream of the *AMY* locus are characterized by unusually low polymorphism in humans when considering the extent of divergence between humans and chimpanzee in the same regions (Fig. 2). The *AMY*-surrounding 50 kb regions belonged to the bottom 1% of the distribution in terms of the lowest cumulative frequency of derived alleles at polymorphic sites and also had a high concentration of derived alleles that are fixed in all human populations while being found in ancestral state both in the high coverage Altai Neanderthal and Denisovan data. The regions surrounding the *AMY* cluster of genes also belong to the small fraction of just 0.28% of the genome-wide 50 kb regions that in the 1000 Genomes sequence data^21^ have a coalescent date which is younger than the human-Neanderthal population split, estimated at 650 KYA^18^,^22^, and are characterized by the presence of multiple fixed derived alleles in all humans, which are homozygous and in ancestral state both in the high coverage Altai Neanderthal and Denisovan genomes. Altogether, these findings suggest that human *AMY* genes cluster in a genomic neighbourhood which has undergone a selective sweep in human ancestry following the split from the Neanderthals.

**Figure 3:**
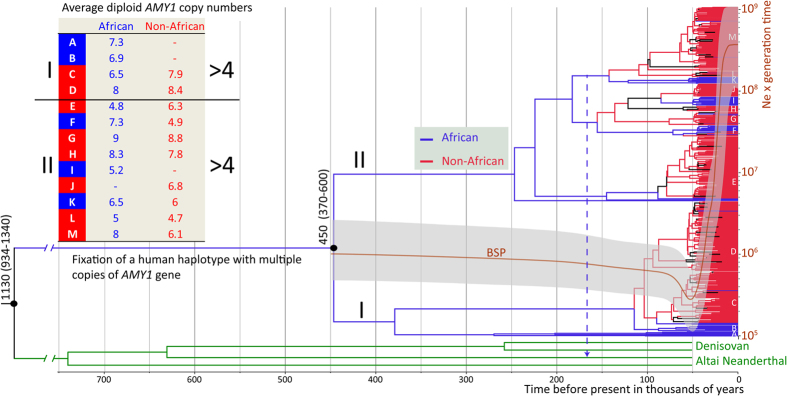
Phylogenetic tree and Bayesian Skyline Plot based on the analyses of a ~66 kb region downstream of the AMY locus with BEAST. The analyses were restricted to bi-allelic SNP variants from a ~66 kb long high LD region (Chr1:104,303,310-104,369,301) which is downstream of (centromeric to) *AMY1C* gene (Figure S1). Coalescent time estimates are shown near branching points and assume mutation rate of 5.5 × 10^−10^ per bp per year^34^. BSP–Bayesian Skyline Plot; thick brown line shows the median estimates of human effective population size (N_e_) x generation time (y-axis) over time (x-axis) as estimated from the ~66 kb region data in the global sample of 480 individuals. The grey shaded area around the brown line shows the 95% higher posterior density intervals of the BSP estimates. A dotted blue arrow highlights the branch of the human phylogeny that has been the likely source of an introgression of a chunk of ~16 kb (Chr1:104,350,432-104,366,871) into the genome of the Altai Neanderthal.

To further study the distribution of genetic variation at the *AMY* locus we subjected a 66 kb sequence from a high LD region, directly downstream of the *AMY1* genes, to phylogenetic analyses using BEAST (Fig. 3). These analyses confirmed the existence of 21 sites where the derived allele is fixed in all humans while the ancestral allele is fixed in archaic humans. Both Altai Neanderthal and Denisovan sequences form a branch that shares a common ancestor with the human lineage at 1130 (95% CI 934–1340) KYA. We estimate the coalescent time of all human lineages at 450 (95% CI 370–600) KYA which postdates the population split of archaic and modern humans at 550–765 KYA^18^,^22^. Consistent with a previous report of ancient human introgression into Neanderthals^23^ we find a short chunk of sequence (Chr1:104,350,432–104,366,871) in the Altai Neanderthal genome which contains, in heterozygous state, derived alleles at four sites out of 11 defining a branch in the phylogeny that is ancestral to human haplotypes K, L and M. These haplotypes have a combined frequency of 24% in our global sample. Because none of the SNPs within this introgressed region that are private to K, L and M haplotypes were found to be shared with the Neanderthal it is likely that the donor haplotype was a distant relative of the K, L and M haplotypes rather than any one of them. The fact that human E-M haplotypes (Fig. 3) are associated each on average with >4 *AMY1* copies per diploid genome makes it likely that at least one of the distant ancestors of the Altai Neanderthal also carried multiple copies of the *AMY1* genes.

In contrast to the finding of human introgression to the Altai Neanderthal genome, none of the 480 individuals from the global set we have sampled showed evidence of either Neanderthal or Denisovan haplotypes, with evidence of a shared haplotype defined by the co-presence of derived alleles at two or more SNPs. The 16 individuals in our global sample, including 2 African and 14 non-African individuals, who carry the ancestral copy number of two *AMY1* genes are therefore unlikely to be explained by the Neanderthal introgression. Furthermore, the sequences of these 16 individuals are not phylogenetically clustered as would be expected if they had low copy number due to the retention of an ancestral haplotype. Instead, they are associated with different haplotypes (D, E, F, I, and M) in the human phylogeny and cluster closely together with lineages characterized by higher *AMY1* copy number in extant populations. The Bayesian Skyline Plot analysis (Fig. 3) reveals a notable drop of effective population size (Ne) at the AMY locus between 80–50 KYA followed by a major increase in global *N*_*e*_ consistent with the Out-of-Africa expansion. Given the finding of multiple copies of *AMY1*, typically higher than 4 per genome, being characteristic of all main branches of the phylogenetic tree, among African and non-African populations (Fig. 3) it is likely that human populations expanding out of Africa already carried multiple copies of the *AMY1* genes per chromosome and the finding of occasional cases of two copies per diploid genome are due to secondary losses of *AMY1* copy numbers rather than retention of the ancestral state at this locus. Apart from a small number of low frequency haplotypes that are by and large restricted to African (A, B, I, and K) or non-African (J, L) populations all common haplotypes (C-H, M), together accounting for 90% of the global variation, are widely spread in all major continental regions (Table 2, Tables S1 and S2).

**Figure 4:**
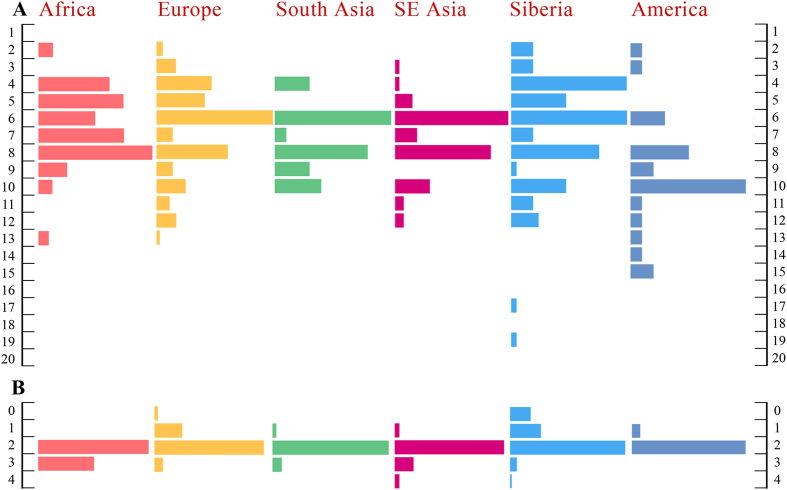
Distributions of *AMY1* and *AMY2A* copy number across the major continental groups.

The AMY copy numbers referred to above were based on the inferences made from the read depths in the high coverage whole genome sequences. The full set of AMY copy number predictions for the global set of 480 human high coverage sequences are presented in Table S1. We estimated the accuracy of the read depth based inference of AMY copy number using high-resolution DNA typing methods^3^ in a subset of 68 samples. We observed Pearson’s correlation coefficient r = 0.92 between the copy number predictions from read depth data and the paralogue ratio assay results for *AMY1*, and 0.85 for *AMY2A* (Table S1). At the same time, we observed errors up to 70% for making exactly the right inference of *AMY1* copy when copy numbers were higher than 10, e.g. distinguishing 11 copies from 12. For *AMY1* results correlation was at 92% for copies between 2 and 7 but dropped to 75% at copy numbers higher than this. These results show that we can, with reasonably high confidence, estimate the copy number of amylase genes from read depth data whereas the accuracy of distinguishing exact copy numbers in individual cases can be quite low. Nevertheless, consistent with previous studies based on accurate methods of AMY CNV inference^3^,^4^ we observe significant bias in our read depth based data towards even copy numbers of *AMY1* across the globe (355 even versus 125 odd calls) in all regional populations (Fig. 4B). The excess of even copy number diploid genotype calls is a consequence of haplotypes having predominantly odd number of *AMY1* copies. The parity between simultaneously even *AMY1* and *AMY2A* copy numbers is also reproduced here, along with the global average *AMY1* copy number of 7.1 in line with previously published mean read depth of 7.4 copies in the HapMap^4^ and 7.3 in the 1000 Genome Project data^3^.

Pancreatic and salivary amylase genes show different ranges of copy number variation. While the minimum number of salivary *AMY1* copies (Fig. 4B) we observed in our global sample was 2, in the case of pancreatic amylases we find a number of individuals (Fig. 4A, Table S1) with just a single or no copies of the *AMY2A* gene. However, all individuals sampled (Tables S1 and S2) carry at least two pancreatic copies of the *AMY2B* gene. We observe the highest concentration of the *AMY2A* deletion in Siberia where among the Northeast Siberians it reaches allele frequencies as high as 52% (Table 3). A large proportion (75/86) of the chromosomes carrying the *AMY2A* deletion world-wide is clustered within a restricted number of haplotypes, with haplotypes L and M accounting for the highest share (Table 3). These haplotypes are associated with derived alleles at SNP positions rs1930212 and rs72694406 and CNV-haplotype AH2 that has an even number (2 copies) of *AMY1*, a single copy of *AMY2B* and a deletion of the *AMY2A* copy^4^. Although the direct inference of the phase of *AMY1* CNV-haplotype from our short-read data is not possible the presence and wide geographic spread of the same AH2 copy number haplotype in our data is supported by the observation that 34 out of the 40 (85%) carriers of the *AMY2A* deletion who are heterozygous for the rs72694406 SNP (Table S1) carry an odd number of *AMY1* copies per diploid genome while in contrast the frequency of carriers of odd *AMY1* copy number among individuals who are homozygous for the ancestral rs72694406-G allele is only 19.4%. These results suggest that majority of the *AMY2A* deletions in Europe, Central Asia, and Siberia has a single origin. High frequency of the *AMY2A* deletion in Siberians is also coupled with a notable deficit of individuals with pancreatic amylase duplications relative to populations from lower latitudes (e.g. South and Southeast Asia). Consistent with previous studies^3^,^4^ we find that the majority (24/25) of the duplication of both *AMY2A* and *AMY2B* genes occur on the background of rs12075086-T and rs79043596-C alleles that define haplotypes I and J (Table S1) which are common in African and West Eurasian populations, respectively (Table 2). We observe weak but significantly negative correlations with latitude both for *AMY2A* and *AMY1* copy numbers (Table 4). These correlations remain highly significant after the exclusion of the Northeast Siberian group with the highest *AMY2A* deletion frequency (data not shown). Similarly to the trends observed previously in dog *AMY2B* copy number data^24^ we find that human populations from higher latitudes also show higher variance of amylase copy numbers (Table 2).

## Discussion

In this study we have analysed genetic regions surrounding the human AMY cluster for evidence of natural selection and we have found: that human populations within and outside Africa are characterized by unusually low genetic diversity in the flanks of amylase locus relative to other genetic loci genome-wide; a young coalescent date postdating the human-Neanderthal population split; a significant Tajima’s D signal in Africans; and the lack of strong signal of recent positive selection in human population groups we studied. These results are generally in line with Middle Pleistocene^9^ rather than Holocene^1^ selection at the *AMY* locus although the significantly negative correlations between amylase copy numbers and latitude (Table 4), and the lower variance at low latitudes (Table 2) may point to the effect of long term and potentially recent selection that has maintained the copy numbers in populations relying on starch-based diets. Our finding that all human lineages at this locus coalesce to one ancestral lineage after the human-Neanderthal split, together with the evidence of both Neanderthals and Denisovans having the ancestral set of just two *AMY1* copies^18^ gives more credence to the scenario that ties the multiplication of amylase copy numbers in association of a selective sweep specifically to the human ancestors^25^, rather than within the species ancestral to both humans and Neanderthals^9^. It also suggests longer food processing sequences, for example involving grinding, leaching and cooking of starch rich tubers in the middle and later Middle Pleistocene.

Our phylogenetic inferences suggest that people moving out of Africa already carried multiple copies of the *AMY1* gene. The genome of the 45 KYA Ust’Ishim man from Siberia^26^ is homozygous for all the four SNPs that define haplotype M (Fig. 3). The core sequence of haplotype M shared by the Ust’Ishim man is identical over the 66 kb sequence range with haploid sequences from 60 modern individuals who carry on average 6.15 copies of *AMY1* per diploid genome. The genome of the Mesolithic European from Loschbour has been previously^2^ estimated to carry 13 copies of *AMY1*. The analysis of SNP variation in the 66 kb region reveals that the Loschbour individual was heterozygous for SNPs defining haplotypes C and D, carriers of which have a combined haplotype frequency of 31.5% in present-day Western Europe with an average *AMY1* copy number of 7 per diploid genome (Tables S1 and S2). The Neolithic farmer from Stuttgart, estimated to carry 16 *AMY1* copies, is homozygous for three SNPs defining the G haplotype which has 8% frequency in present-day Europe. Individuals in the 1000 Genome Project data who are homozygous for the haplotype G defining rs74344448-C allele (Table S2) carry on average 10 copies of *AMY1*. So, for both the Mesolithic and Neolithic aDNA samples where the *AMY1* copy number has been estimated the copy numbers in their related lineages show a trend in present-day populations to have decreased rather than increased on average over time.

A high range of variation in amylase copy numbers in present-day human populations could result from both copy number increases as well as deletions. The high frequency of *AMY2A* deletion we observed in our Siberian sample could be an outcome of neutral and/or selective processes. The possibility of neutrality is raised by the small long term effective population size of Northeast Siberians^27^, which implies high levels of genetic drift. Haplotypes containing deletions of the pancreatic *AMY2A* could have risen in frequency through random fluctuations in the absence of starch-digestion related selection. Such a relaxation in selective maintenance is supported by our observation that populations of higher latitudes are characterized by higher variance of amylase copy numbers (Table 2) and the dietary ecology argument: the diet of Siberian populations that have historically followed migrating herds of reindeer and woolly mammoth would have included relatively limited plant carbohydrates. A diet focussed on reindeer and woolly mammoth would have provided significant quantities of fat and hence the lipids needed, in the absence of bulky plant carbohydrates, to offset the nitrogen toxicity associated with a high protein diet^28^. One of the strongest selective sweeps in the human genome, the mutation of the *CPT1A*, involved in the oxidation of long-chain fatty acids, attests to this ecological adaptation in circum-Arctic populations^27^. Archaeological evidence for plant diet at these time depths is limited and the lower *AMY2A* numbers found in these regions suggest a less important role of starchy plants than in many other populations. Archaeological research in the Yana Valley (27 KYA) excavations concluded those who camped at the site hunted reindeer, horses and birds^29^. Archaeobotanical investigations at Dolní Vĕstonice II, Czech Republic, date to 30 KYA, at a latitude below the boreal zone, revealed extensive exploitation of dietary roots and tubers^30^ in addition to the hunting of reindeer and mammoth.

In conclusion, we find evidence for unusually low sequence diversity in regions flanking the amylase copy number locus in a global set of human populations. Significantly negative Tajima’s D scores in Africans, the presence of fixed differences between modern and archaic humans, and young coalescent date in the modern human lineage together suggest that the amylase locus has undergone a selective sweep after the separation of humans and Neanderthals. It is likely that this selective sweep that fixed multiple copies of *AMY1* gene in modern human lineages was associated with a dietary shift and an elaboration of the processing sequences for starch-rich tubers, incorporating, for example, grinding, leaching and cooking.

## Methods

We assessed the distribution of genetic diversity near the *AMY* locus from the Phase 1 of the 1000 Genomes panel^21^. Genetic diversity at the *AMY* locus was placed in the context of the patterns of genetic diversity observed in the rest of the genome by 50 kb non-overlapping windows. We applied the 1000 Genomes Project’s accessible genome strict mask (release 20141020) to retain information only from regions that can be uniquely mapped by Illumina short reads. As a consequence of applying these filters, 50 kb windows displaying less than 40 kb of accessible genome with available ancestral information were removed from downstream analyses. We used the Human-Chimpanzee ancestral sequence chimpanzee genome (inferred from the Sequences in Ensembl v64 EPO Compara 6 primate block^18^) as an outgroup and focused on two parameters: the cumulative frequency of derived alleles over polymorphic sites in Africans in a 50 kb window and the number of fixed derived sites in Africans where high coverage Altai Neanderthal^18^ and Denisovan^31^ sequences both carry the ancestral allele. The first of these parameters was estimated to serve as a proxy of the coalescent age of a given 50 kb window in humans while the second parameter was designed to distinguish cases of complete and incomplete lineage sorting among modern and archaic humans. We chose to focus on African genomes only, rather than the full 1000 Genomes panel, to minimize the effect on these statistics of archaic admixture that has been documented in non-Africans^18^,^32^.

To estimate the age of the haplotypes associated with ancestral copy number of the *AMY1*, *AMY2A* and *AMY2B* genes we used phased high coverage data for 480 whole genome sequences determined with the Complete Genomics platform^19^. We examined firstly the patterns of linkage disequilibrium, using the D’ statistic (Figure S1), in a region of ~100 kb both up and downstream of the *AMY* locus to define the boundaries of a region of extremely high linkage disequilibrium in our global data set for further haplotype based analyses. The D’ statistic was calculated for bi-allelic SNPs that had MAF >0.1 in our global sample. These analyses identified a ~66 kb region (Chr1:104,303,310–104,369,301) downstream of the AMY locus with high average (D’ = 0.992) LD among SNPs. Notably, the SNPs in the ~66 kb region show also relatively high average (D’ = 0.839) LD with the SNPs from the 150 kb region (Chr1:104,000,001–104,150,000) upstream of the AMY copy number variable locus, suggestive of a strong long-range association. We determined the phylogenetic relationships and ages of ancestral haplotypes in the ~66 kb region using BEAST version 1.8^33^. In all age calculations we used the mutation rate of 5.5 × 10^−10^/bp/year^34^ with the relaxed lognormal clock and piecewise-linear Bayesian Skyline model with 10 groups. Maximum Likelihood trees inferred with RAxML v. 7.8.6^35^ were provided as starting trees. Eight independent BEAST analyses were run for 100 million iterations, sampling every 5,000 steps. After inspection in Tracer v1.6^33^, the results from the independent runs were merged using LogCombiner v1.8.0 with a burn-in of 20%. Then the Bayesian Skyline Plots were reconstructed in Tracer, confirming that the ESS values were above 200.

To infer the copy numbers of *AMY1, AMY2A* and *AMY2B* genes, we used high coverage sequence data for 480 individuals from 125 populations sampled worldwide^19^. A subset of 68 DNA samples from Siberia, Southeast Asia and the Andes were further subjected to paralogue ratio test and microsatellite analyses for determining copy number variation in the *AMY* locus using methods described elsewhere^3^. Informed consent had been obtained from all human subjects tested here, and the genome-scale work on their DNA had been approved by the ethics committees of the Institute of Biological Problems of the North of the Russian Academy of Sciences in Magadan (statement no. 001/011 from January 21, 2011) and by the AMIS-UPS Research Ethics Committee, University of Toulouse, Paul Sabatier (Ethical approval no. 005/011). All genetic analyses and data manipulations were performed in accordance with the relevant guidelines approved by the Cambridge Human Biology Research Ethics Committee (HBREC.2011.01).

To determine the *AMY1*, *AMY2A* and *AMY2B* copy numbers from the read depth data we used the ‘relative coverage’ (R) metric reported by CG. Considering the fact that the reference genome contains three haploid copies of *AMY1* genes (*AMY1A, AMY1B, AMY1C*) the combined *AMY1* copy number per diploid genome was determined by (R_*AMY1A*_/R_*AMY2B*_) × 6. This method yielded a Pearson correlation coefficient r = 0.92 with the results based on *AMY1* paralogue ratio test. All 68 samples tested had two copies of *AMY2B* by the read depth and paralogue ratio tests. *AMY2A* copy numbers inferred from the CG output showed Pearson correlation coefficient r = 0.85 with the assay results.

In order to reveal the haplotypes on the background of which *AMY2A* deletion occurs (Table 3) we determined the phase of the *AMY2A* deletions in the context of the phased data for 891 binary SNPs in the Chr1:104,303,310–104,369,301 (hg19) region. Firstly, we estimated the genotype of *AMY2A* deletion for each sequenced individual by subtracting the read-depth inferred number of *AMY2B* copies from the *AMY2A* copy number. We assigned samples with equal or higher number of *AMY2A* copies as homozygous non-carriers of the deletion, individuals with single difference as heterozygotes and individuals with two more copies of *AMY2B* as homozygotes for the deletion. Beagle 4.1^36^ was used to phase the heterozygous samples and to impute samples with missing *AMY2A* information.

## Additional Information

**How to cite this article**: Inchley, C. E. *et al.* Selective sweep on human amylase genes postdates the split with Neanderthals. *Sci. Rep.* **6**, 37198; doi: 10.1038/srep37198 (2016).

**Publisher’s note:** Springer Nature remains neutral with regard to jurisdictional claims in published maps and institutional affiliations.

## Supplementary Material

Supplementary Information

Supplementary Tables

## Figures and Tables

**Table 1:** Genome-wide significance of the window containing AMY genes by three selection tests in 12 regional population groups.

AMY WINDOW (Chr1 104-104.2 Mb)	iHS	nSL	TD
Africa	0.99	0.97	0.0024**
Middle East	*0.02*>*	0.12	*0.03*>*
South & West Europe	0.08	0.25	0.07
East & North Europe	0.93	0.06	0.08
Volga Uralic	0.19	0.28	0.14
South Asia	0.16	0.23	*0.04*>*
West Siberia	0.88	0.41	0.24
South Siberia & Mongolia	0.21	0.10	0.11
Central Siberia	*0.03*>*	0.12	0.19
Northeast Siberia	0.38	0.36	0.06
Southeast Asia Mainland	0.91	0.19	0.08
Island Southeast Asia	0.06	*0.05*>*	0.18

^*^Indicates cases where the AMY genes containing window had the relevant selection test score ranking in the top 5% of the windows (p < 0.05), bold

^**^indicates cases where the AMY windows was within the top 1% of windows for the given positive selection test (p < 0.01).

**Table 2:** Geographic distribution of haplotypes in the Chr1:104,303 K-104,369 K region.

	N individuals	Average	Average diploid copy number (σ^2^)			Haplotype distribution (Heterozygote counting as 1, homozygote as 2 copies)												
		|latitude|	*AMY1*	*AMY2A*	*AMY2B*	A	B	C	D	E	F	G	H	I	J	K	L	M
Africa	33	3.6	6.4 (3.4)	2.3 (0.2)	2.1 (0.1)	5	10	4	3	4	8	4	3	12		11		2
West Asia/Caucasus	59	39.2	7.2 (6.2)	2.2 (0.5)	2.3 (0.3)			15	25	13	3	10	6		25			21
Southwest Europe	23	47.3	6.5 (3.9)	1.8 (0.2)	2 (0)			6	5	13		4			6		1	11
Northeast Europe	80	57.3	6.5 (6.5)	1.8 (0.3)	2.1 (0)			25	40	36	8	9	5		11		4	22
Volga-Ural region	25	54.9	7.4 (5.8)	1.7 (0.6)	2 (0)			10	10	11		3			3		3	10
South Asia	29	23	7.2 (3.3)	2.2 (0.3)	2.1 (0.2)			8	16	20			1		2			11
Central Asia	24	40.9	7.6 (6.5)	2.2 (0.5)	2.1 (0.2)			9	7	12		2			5	1		12
West Siberia	18	63.7	5.9 (5.9)	1.4 (0.6)	2 (0)			8	6	9	2	1					2	8
South Siberia	34	52.8	7.6 (4.2)	1.9 (0.3)	2 (0)			16	20	15			1				2	14
Central Siberia	27	64.1	6.5 (13)	2.1 (0.3)	2 (0)			11	9	16					2			16
Northeast Siberia	23	62.6	6.2 (14.8)	1 (0.6)	2 (0)			1	7	9								29
South America	28	24.2	9.3 (9.3)	1.9 (0.1)	2 (0)			10	31									15
East Asia	26	22.9	7.7 (4.3)	2 (0)	2 (0)			12	20	11								9
Island Southeast Asia	45	9.4	7.1 (2.8)	2.1 (0.1)	2 (0)			19	26	16					1			28
Papua New Guinea	6	7	6.3 (0.3)	2.5 (0.3)	2 (0)					12								
Total	480	39.5	7.1 (6.5)	1.9 (0.4)	2.1 (0.1)	5	10	154	225	197	21	33	16	12	55	12	12	208
						0.01	0.01	0.16	0.23	0.21	0.02	0.03	0.02	0.01	0.06	0.01	0.01	0.22

**Table 3:** Geographic distribution of the AMY2A deletion.

		N	*AMY2A* deletion		Haplotype distribution of *AMY2A* deletion by haplotypes												
			count	frequency	A	B	C	D	E	F	G	H	I	J	K	L	M
1	Africa	36	0	0.00													
2	West Asia/Caucasus	110	5	0.04			1										4
3	South Asia	58	1	0.02													1
4	Southwest Europe	46	5	0.11			1		1							1	2
5	Northeast Europe	158	16	0.10			2									3	11
6	Central Asia	48	4	0.08			1										3
7	Volga Ural region	44	8	0.18			1									3	4
8	West Siberia	36	10	0.28			1									2	7
9	South Siberia	68	6	0.09			1									2	3
10	Central Siberia	54	3	0.06													3
11	Northeast Siberia	46	24	0.52				1									23
12	South America	56	2	0.04													2
13	East Asia	52	1	0.02													1
14	Island Southeast Asia	90	1	0.01					1								
15	Papua New Guinea	6	0	0.00													
				Deletions	0	0	8	1	2	0	0	0	0	0	0	11	64
				Total count	2	4	151	218	195	20	30	15	4	54	11	11	199
				Deletion frequency	0.00	0.00	0.05	0.00	0.01	0.00	0.00	0.00	0.00	0.00	0.00	1.00	0.32

Note: N – number of phased chromosomes with information for *AMY2A* and *AMY2B* copy numbers.

**Table 4:** Correlations between amylase copy numbers and geographic latitude and longitude.

Spearman rank-order	Copy number of		
correlation coefficients r_s_	*AMY1*	*AMY2A*	*AMY2B*
Absolute latitude	−0.19 (p = 2 × 10^−5^)	−0.33 (p < 10^−6^)	−0.05 (p = 0.29)
Longitude	0.001 (p = 0.98)	0.001 (p = 0.98)	−0.098 (p = 0.035)
*AMY2A* copy number	0.21 (p = 9 × 10^−6^)		
*AMY2B* copy number	−0.15 (p = 0.0015)	0.48 (p < 10^−6^)	

Note significance for the two-tailed Spearman rank-order correlation test is shown in parenthesis.
