# Supplementary material for: Selective sweep on human amylase genes postdates the split with Neanderthals
Source: Sci Rep. 2016 Nov 17;6:37198. doi: 10.1038/srep37198 (PMC5112570; doi:10.1038/srep37198)
Supplement: Supplementary Information [file srep37198-s1.pdf]

Selective sweep on human amylase genes postdates the split with Neanderthals

Charlotte E. Inchley[1*], Cynthia D.A. Larbey[1], Nzar A.A. Shwan[2], Luca Pagani[1,3], Lauri Saag[3], Tiago Antão[4], Guy Jacobs[5], Georgi Hudjashov[3,6], Ene Metspalu[3], Mario Mitt[7,8], Christina A. Eichstaedt[1,9], Boris Malyarchuk[10], Miroslava Derenko[10], Joseph Wee[11], Syafiq Abdullah[12], François-Xavier Ricaut[13], Maru Mormina[14], Reedik Mägi[8], Richard Villems[3], Mait Metspalu[3], Martin K. Jones[1], John A. L. Armour[2], Toomas Kivisild[1,3*]

[1] Department of Archaeology and Anthropology, University of Cambridge, Cambridge, CB2 3QG, UK

[2] School of Life Sciences, Queen's Medical Centre, University of Nottingham, Nottingham NG7 2UH, UK

[3] Estonian Biocentre, Tartu, 510104, Estonia

[4] Division of Biological Sciences, University of Montana, Missoula, MT, USA

[5] Complexity Institute, Nanyang Technological University, Singapore

[6] Statistics and Bioinformatics Group, Institute of Fundamental Sciences, Massey University, Palmerston North, New Zealand

[7] Estonian Genome Center, University of Tartu, Tartu, Estonia

[8] Department of Biotechnology, Institute of Molecular and Cell Biology, University of Tartu, Tartu, Estonia

[9] Thoraxclinic at the University Hospital Heidelberg, 69126 Heidelberg, Germany

[10] Genetics Laboratory, Institute of Biological Problems of the North, Russian Academy of Sciences, Magadan, Russia

[11] Division of Radiation Oncology, National Cancer Centre, Singapore, Singapore;

[12] RIPAS Hospital, Bandar Seri Begawan, Brunei Darussalam

[13] Evolutionary Medicine group, Laboratoire d'Anthropologie Moléculaire et Imagerie de Synthèse, UMR 5288, Centre National de la Recherche Scientifique, Université de Toulouse 3, Toulouse, France

[14] Department of Applied Social Sciences, University of Winchester, Sparkford Road, Winchester SO22 4NR, UK

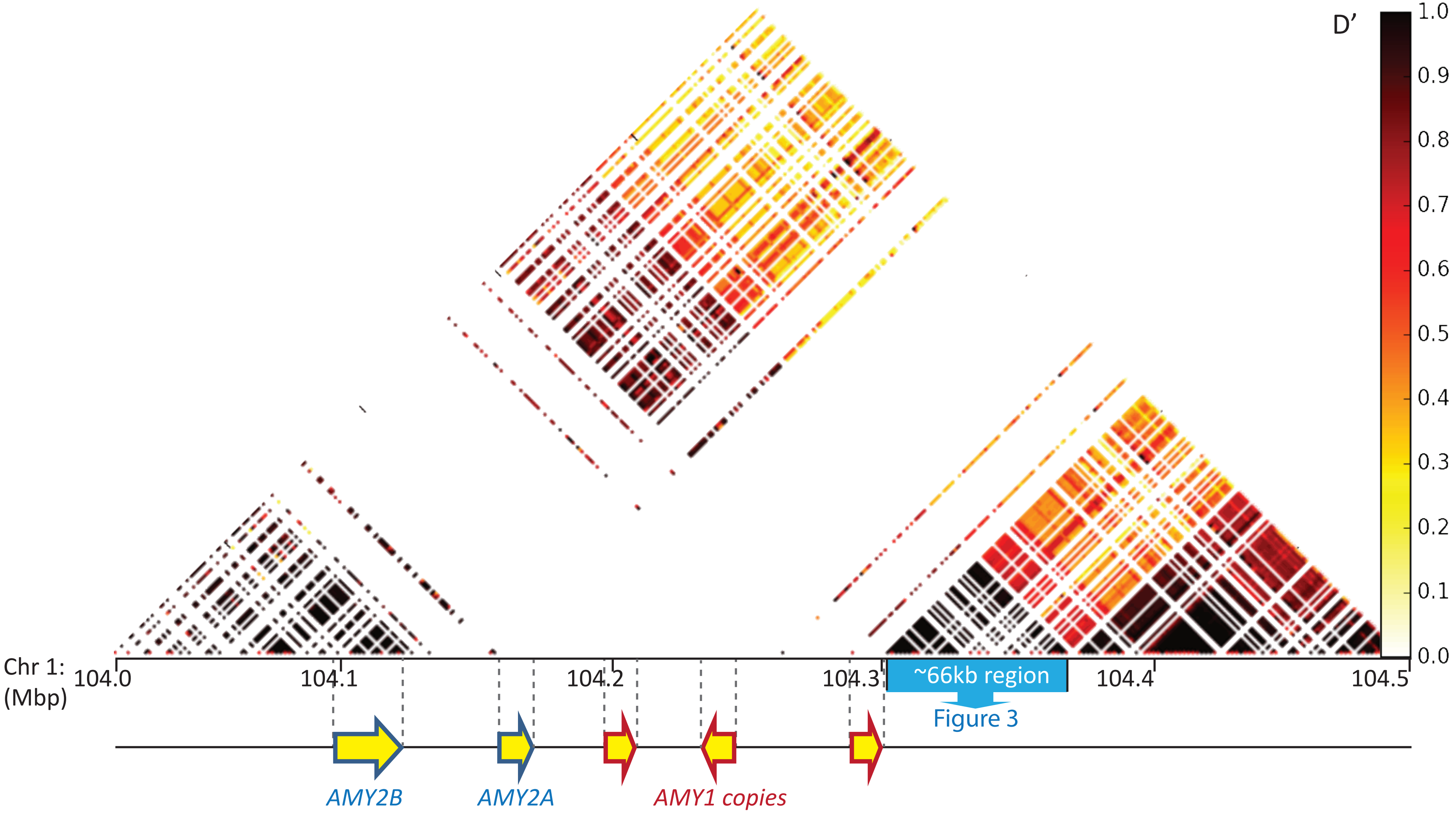

Figure S1. Distribution of D’ estimates of linkage equilibrium in the 3’ and 5’flanks of the amylase copy number variable locus.
The plot is restricted to bi-allelic SNPs from a 0.5 Mb region of chromosome 1 (Chr1:104,000,000-104,500,000) and shows estimates of LD for SNPs with MAF >0.1 in the global data. Yellow arrows indicate the location of the amylase genes. The gap of SNP data from the copy number variable region is due to the difficulty of mapping short reads in regions of high sequence homology. The ~66kb high LD region (Chr1:104,303,310-104,369,301), further used in the phylogenetic analyses (Figure 3), is highlighted in blue.
